# Drought Resistance by Engineering Plant Tissue-Specific Responses

**DOI:** 10.3389/fpls.2019.01676

**Published:** 2020-01-22

**Authors:** Damiano Martignago, Andrés Rico-Medina, David Blasco-Escámez, Juan B. Fontanet-Manzaneque, Ana I. Caño-Delgado

**Affiliations:** Department of Molecular Genetics, Centre for Research in Agricultural Genomics (CRAG) CSIC-IRTA-UAB-UB, Barcelona, Spain

**Keywords:** drought, *Arabidopsis*, cereals, genome editing, cell-specific regulation

## Abstract

Drought is the primary cause of agricultural loss globally, and represents a major threat to food security. Currently, plant biotechnology stands as one of the most promising fields when it comes to developing crops that are able to produce high yields in water-limited conditions. From studies of *Arabidopsis thaliana* whole plants, the main response mechanisms to drought stress have been uncovered, and multiple drought resistance genes have already been engineered into crops. So far, most plants with enhanced drought resistance have displayed reduced crop yield, meaning that there is still a need to search for novel approaches that can uncouple drought resistance from plant growth. Our laboratory has recently shown that the receptors of brassinosteroid (BR) hormones use tissue-specific pathways to mediate different developmental responses during root growth. In *Arabidopsis*, we found that increasing BR receptors in the vascular plant tissues confers resistance to drought without penalizing growth, opening up an exceptional opportunity to investigate the mechanisms that confer drought resistance with cellular specificity in plants. In this review, we provide an overview of the most promising phenotypical drought traits that could be improved biotechnologically to obtain drought-tolerant cereals. In addition, we discuss how current genome editing technologies could help to identify and manipulate novel genes that might grant resistance to drought stress. In the upcoming years, we expect that sustainable solutions for enhancing crop production in water-limited environments will be identified through joint efforts.

## Introduction

Today, agriculture is facing an unprecedented challenge. Arable land is being reduced by soil erosion and degradation, desertification, and salinization, destructive processes that are being further accelerated by climate change. This could jeopardize global food production, which will need to be maximized to cope with the world´s growing population and to match the food security goals established by United Nations. More than ever, drought is a major threat to agriculture worldwide. The Food and Agriculture Organization (FAO) of the United Nations documented that between 2005 and 2015, drought caused USD 29 billion in direct losses to agriculture in the developing world, with the 2008–2011 drought in Kenya alone accounting for USD 1.5 billion (FAO, 2018). In addition, more than 70% of the world´s available fresh water is being used in irrigation (Organization for Economic Cooperation and Development, 2017). To cope with these challenges, plant breeders will need to begin producing novel crop varieties that have increased yield, that are tolerant to abiotic stresses, and that have improved water and nutrient uptake efficiencies (Fita et al., 2015).

In agronomy, drought can generally be defined as a prolonged lack of water that affects plant growth and survival, ultimately reducing crop yield. In plant science, the broadest definition of drought stress coincides with the definition of water deficit, which happens when the rate of transpiration exceeds water uptake (Bray, 1997). This could be the result of a lack of water, but also of increased salinity or osmotic pressure. From a molecular biology perspective, the first event during drought stress is the loss of water from the cell, or dehydration. Dehydration usually triggers signals that are osmotic and hormone related, with abscisic acid (ABA) mainly involved in the latter (Blum, 2015). These signals are followed by a response that could be broadly categorized into three main strategies: i) drought escape (DE), ii) dehydration avoidance, and iii) dehydration or desiccation tolerance (Kooyers, 2015; Blum and Tuberosa, 2018). DE is the attempt of a plant to accelerate flowering time before drought conditions hinder its survival. This response is common to annual plants including the model species *Arabidopsis thaliana* (*Arabidopsis*), and is exploited by cereal plant breeders (Shavrukov et al., 2017). In dehydration avoidance, the plant is able to maintain a high relative water content (RWC% = [fresh mass − dry mass]/[water saturated mass − dry mass] × 100) even during water scarcity. This is achieved by physiological and morphological responses that include the reduction of transpiration *via* ABA-mediated stomatal closure, the deposition of cuticular waxes, and the slowing down the plant´s life cycle. Dehydration avoidance usually leads to survival through delaying plant growth, and thus senescence and mortality. This strategy evolved as a response to moderate, temporary drought stress in which the plant undergoes a developmental stand-by until the next rainfall (or irrigation). While effective in increasing plant survival rate, dehydration avoidance often comes with growth and yield penalties, which are, of course, major negative traits for crop breeders (Skirycz and Inzé, 2010). On the other hand, in dehydration tolerance, the plant is able to maintain its functions in a dehydrated state, usually by regulation of plant metabolism to increase the production of sugars, osmoprotectants, antioxidants, and reactive oxygen species (ROS) scavengers (Hu and Xiong, 2014). These responses are usually activated by gibberellic acid (GA) signaling through the modulation of the GA-signaling molecule DELLA, a pathway that integrates multiple hormone- and stress-related pathways (Vandenbussche et al., 2007; Navarro et al., 2008; Colebrook et al., 2014).

Ultimately, drought resistance is determined by how a plant efficiently and timely senses changing environmental conditions, adopting and combining the aforementioned strategies in response to diminished water availability. Plant breeders have identified physiological traits that result from drought responses and contribute to the adaptation of plants in water-limited conditions. Understanding the molecular and physiological mechanisms behind these traits is essential for improving crops through biotechnology.

In this review, we describe some of the drought resistance traits of the model plant *Arabidopsis* that have the potential of being transferrable to crops, focusing on strategies that involve the manipulation of cell- and tissue-specific responses. As these strategies open up opportunities to uncouple drought resistance from the commonly associated growth and yield penalties, we will discuss their biotechnological application in cereal species.

## Major Traits Contributing to Drought Resistance

### Early Flowering and Drought Escape

The molecular control of flowering time is complex, and has been highly studied in *Arabidopsis* (Michaels and Amasino, 1999; Simpson and Dean, 2002) as well as in many other plant species (Corbesier et al., 2007). During the developmental switch from the vegetative to the reproductive stage, the photoperiodic light signal from the environment is perceived by leaves, where the FLOWERING LOCUS T (FT) protein is synthesized. FT is loaded into the phloem and transported to the shoot apical meristem (SAM) where it initiates floral transition (Andrés and Coupland, 2012). It is now known that in the SAM, FT forms a complex with the bZIP protein FD in specific cells beneath the tunica layers in which FD is expressed, with these cells then originating the floral primordia (Abe et al., 2019).

**Figure 1:**
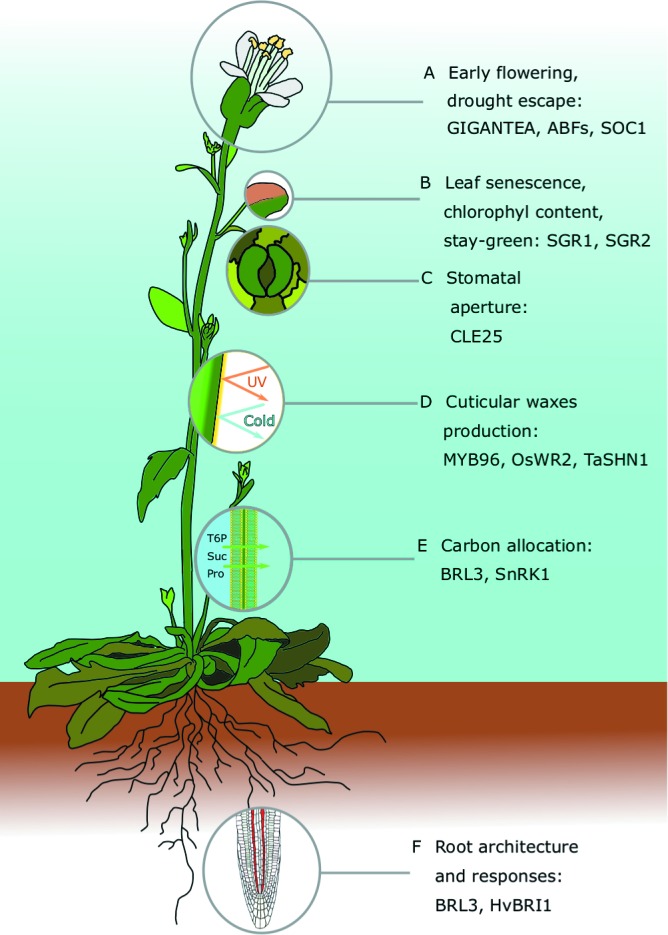
Major traits contributing to drought resistance in *Arabidopsis thaliana:* For each of the traits, we highlight recent and comprehensive review papers, and prominent articles discussed in the main text. **(A)** Early flowering and drought escape: Conti, 2019—spotlight article about the latest discoveries in drought escape; Riboni et al., 2016—relationship between abscisic acid (ABA), GIGANTEA, and flowering time in drought escape; Hwang et al., 2019—molecular mechanisms that allow ABA-responsive element (ABRE)–binding factors (ABFs) to bind to the promoter of the floral master regulator *SOC1* by interacting with a nuclear factor Y subunit C (NF-YC). **(B)** Leaf traits, including senescence, the stay-green trait, and leaf area: Abdelrahman et al., 2017—review about stay-green traits: in *Arabidopsis*, the SGR gene family regulates chlorophyll metabolism during senescence; Distelfeld et al., 2014—review about senescence in cereals. **(C)** Stomatal-mediated drought responses: Bertolino et al., 2019—review about stomatal manipulation toward drought tolerant plants; Papanatsiou et al., 2019—expression of a synthetic K^+^ channel in guard cells improved stomata kinetics and drought avoidance in *Arabidopsis*. **(D)** Cuticular wax production: Lee and Suh, 2015; Xue et al., 2017—reviews about cuticular wax evolution, chemical composition, biosynthesis, and drought responses; Lee et al., 2014—*Camelina sativa* plants overexpressing *Arabidopsis* MYB96 have increased wax biosynthesis and accumulation, and an improved survival rate; Zhou et al., 2014—rice plants that constitutively express the transcription factor *OsWR2* have increased cuticular wax deposition and improved tolerance, but the yield was negatively affected; Bi et al., 2018—wheat plants constitutively expressing *TaSHN1* have altered wax composition, reduced stomatal density, and an improved drought survival rate. **(E)** Carbon allocation: Paul et al., 2018—research update about trehalose 6-phosphate (T6P) and T6P-related approaches to improving crop yield and stress resilience; Griffiths et al., 2016—chemical supply of synthetic analogs of T6P improves dehydration tolerance in *Arabidopsis* and wheat; Fàbregas et al., 2018—overexpression of the *Arabidopsis* brassinosteroid receptor BRL3 improved survival rate by altering carbohydrate distribution in vascular tissues. **(F)** Architecture and response of roots: Rogers and Benfey, 2015—review about root system architecture regulation and possible biotechnological applications in crop improvement; Fàbregas et al., 2018—root vascular expression of AtBRL3 enhanced hydrotropic response of the *Arabidopsis* root.

When *Arabidopsis* is exposed to drought conditions, it can activate the DE response. DE is one of the main defense mechanisms against drought in *Arabidopsis*, and it integrates the photoperiodic pathway with drought-related ABA signaling (Conti, 2019). DE has mainly been studied in an evolutionary context in natural populations (McKay et al., 2003; Franks et al., 2007), and the molecular mechanisms that regulate it have only been unraveled recently. It is known that, to trigger DE, the key photoperiodic gene *GIGANTEA (GI)* needs to be activated by ABA (Riboni et al., 2013; Riboni et al., 2016). A recent breakthrough was the discovery that the ABRE-BINDING FACTORS (ABF) 3 and 4, which act on the master floral gene *SUPPRESSOR OF OVEREXPRESSION OF CONSTANS1 (SOC1)* in response to drought, are involved in this process. The mutants *abf3 abf4* are insensitive to ABA-induced flowering and have a reduced DE response (Hwang et al., 2019). However, the precise molecular mechanisms that link ABA to *GI* and ultimately to DE are still rather obscure, and different crop species might have evolved unknown pathways that trigger DE in different environments (**Figure 1A**).

From an agronomic perspective, DE and early flowering varieties with faster life cycles are interesting because an anticipated switch to the reproductive stage might allow grain filling before the onset of seasonal terminal drought. Furthermore, a shorter crop season reduces the need for agricultural inputs (e.g., fertilizers, pesticides) and might facilitate double cropping (i.e., the farming of two different crops in the same field within the same year). On the other hand, crops that switch too early to flowering will have their yield reduced. Despite DE being an emerging research field in crop science, there are not any biotechnologically improved crops that exploit DE as a drought resistance trait. Still, it has been proposed that DE can be used to obtain quick-growing, early-flowering cereal varieties, which would be especially useful in temperate regions like the Mediterranean area where terminal drought is expected to affect plants toward the end of the crop season (Shavrukov et al., 2017). Furthermore, it has been recently shown that *OsFTL10*, one of the 13 *FLOWERING LOCUS T-LIKE* (*FTL*) genes annotated in the rice genome, is induced by both drought stress and GA, and when overexpressed in transgenic rice plants confers early flowering and improves drought tolerance (Fang et al., 2019). However, as these transgenic rice lines were not tested in a field trial, it is unknown whether engineering *FTL* genes could deliver cereal varieties with superior drought performances and good yield in both dry and well-watered conditions. Nonetheless, the manipulation of the DE pathway could be an innovative and valid strategy especially in the context of highly variable water availability. As DE involves specific tissues (leaf, phloem) and cell types (phloem companion cells, FD-expressing SAM cells), it might be possible to devise strategies aimed at developing drought-resistant plants *via* manipulation of these plant components, adjusting DE to the different environmental conditions.

### Leaf Traits: Senescence, Stay-Green, and Leaf Area

Senescence is a developmental stage of plant leaves that leads to the arrest of photosynthesis, the degradation of chloroplasts and proteins, and the mobilization of nitrogen, carbon, and other nutrient resources from the leaves to other organs. As most cereals are monocarpic annual species, these resources are directed to developing seeds, and senescence therefore plays a relevant role in crop yield. Environmental stresses like temperature, lack of nutrients, and drought might initiate senescence prematurely, affecting seed nutritional composition and crop yield (Buchanan-Wollaston, 1997; Distelfeld et al., 2014). In crops threatened by terminal drought, the ability to sustain photosynthetic activity longer by delaying or slowing down senescence could be an effective strategy to avoid yield losses. As such, leaf senescence has been extensively studied in crops (**Figure 1B**).

**Figure 2:**
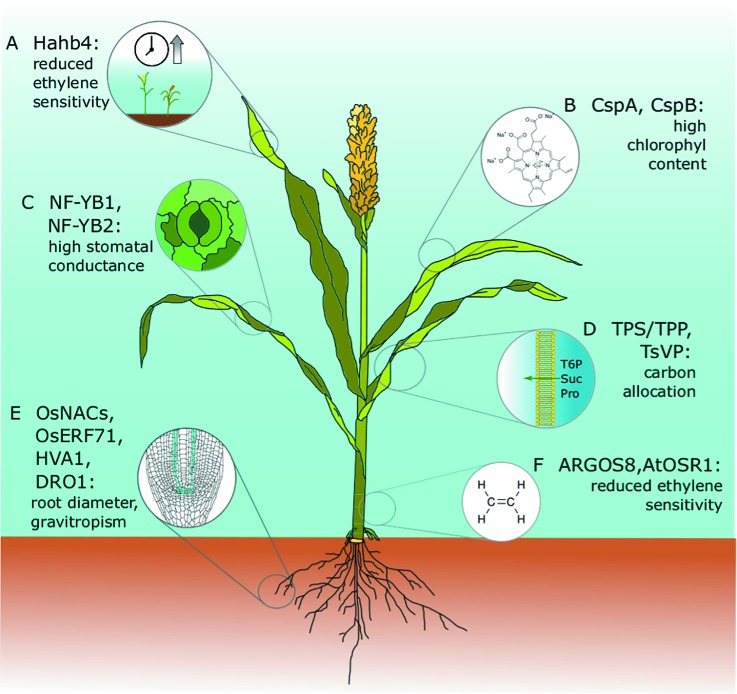
Drought tolerance genes that have been discovered or tested in model species and translated successfully into crop species. All of these genes have been expressed in engineered cereal crops and have been tested in field trials. Major agronomical traits, including yield, have been assessed, and conditions and drought performances have been successfully improved without negatively affecting plant growth or crop yield. **(A)** Hahb4: The sunflower transcription factor Hahb4 was expressed in soybean under the control of the native stress-inducible promoter of a homologous gene. Transgenic plants have reduced ethylene sensitivity, delayed senescence, increased osmoprotectant content, and an increased yield in the presence or absence of drought stress (Waltz, 2015). These plants are currently on the market as Verdeca Drought Tolerance Soybeans HB4^®^. The same Hahb4 has also been transferred to bread wheat under the control of the constitutive promoter of maize ubiquitin 1 with similar promising results (Gonzalez et al., 2019; Ayala et al., 2019). **(B)** CspA, CspB: Maize plants overexpressing *Escherichia coli* CspB have high chlorophyll content, an improved photosynthetic rate, and reduced leaf area during vegetative growth. The best performing lines were commercialized as Genuity^®^ DroughtGard™ by Monsanto (now Bayer) in 2010 (Castiglioni et al., 2008; Nemali et al., 2015). **(C)** NF-YB1, NF-YB2: Maize plants overexpressing ZmNF-YB2 have higher stomatal conductance and chlorophyll content, and delayed senescence. These lines were not assessed in the field for performance under well-watered conditions and were never introduced to the market (Nelson et al., 2007). **(D)** TPS/TPP, TsVP: Carbon allocation, root/shoot ratio. In maize, floral-specific expression of T6P phosphatase (TPP) altered carbon allocation and improved yield in both well-watered and water-limited field trials (Nuccio et al., 2015). Also, in maize, the constitutive expression of the TsVP gene from the halophyte *Thellungiella halophila* under the control of the endogenous ubiquitin promoter increased total soluble sugars and proline under osmotic stress. Improvements in dehydration tolerance were assessed in a small-scale field trial (Li et al., 2008). **(E)** OsNACs, OsERF71, HVA1, DRO1. In rice, expression of the transcription factor OsNAC5 under the control of the root-specific promoter RCc3 improved drought and high salinity resistance by enlarging the root diameter. Yield improvements in normal and stress conditions were assessed in a 3-year field trial in three different locations (Jeong et al., 2013). Similar results were obtained with OsNAC9 and OsNAC10 (Jeong et al., 2010; Redillas et al., 2012). Root-specific expression. Also, in rice, the expression of the barley HVA1 under the control of a synthetic ABA-inducible promoter enhanced root growth, leading to better water use efficiency and abiotic stress tolerance. as confirmed by a small-scale field trial (Chen et al., 2015). The DRO1 allele from deep-rooting rice cultivars increases gravitropic response and root depth, increasing rice yield in both drought and normal conditions (Uga et al., 2013; Arai-Sanoh et al., 2014). **(F)** AtOSR1, ARGOS8: *Arabidopsis* ORGAN SIZE RELATED1 (AtOSR1) 1 and its maize homolog ZmARGOS1 improve dehydration avoidance in both plant species by reducing ethylene sensitivity (Shi et al., 2015); Moderate constitutive expression of ARGOS8, which was obtained by promoter swapping using clustered regularly interspaced short palindromic repeats/CRISPR-associated protein 9 (CRISPR/Cas9) homology-directed recombination, improved drought tolerance in a field trial under stress conditions without affecting yield in well-watered control experiments (Shi et al., 2017). Commercialization of these lines is under evaluation by the developer Corteva Agriscience™ (former DuPont Pioneer).

Plant breeders commonly refer to the trait that confers extended photosynthetic activity as stay-green, also defined as green leaf area at maturity (GLAM). This trait is well studied in sorghum [*Sorghum bicolor* (L.) Moench], a dry climate-adapted cereal in which a number of stay-green quantitative trait loci (QTLs) have been identified (Vadez et al., 2011). However, the genes underlying these QTLs have not yet been identified (Harris-Shultz et al., 2019). Stay-greenness in sorghum is a complex trait, and it is also connected with the perennial tendencies of some varieties (Thomas and Howarth, 2000). Other plant species achieve stay-green characteristics *via* substantially different pathways that include disabling chlorophyll catabolism (like in the case of Gregor Mendel’s green peas, Armstead et al., 2007), and altering the responses to plant hormones. Indeed, some stay-green genes have also been identified in *Arabidops*is and rice (Hörtensteiner, 2009), notably the *Stay-Green Rice* (SGR) genes and their homologs in *Arabidopsis* SGR1, SGR2, and SGR-like (SGRL). The respective molecular pathways have been elucidated, with the phytohormones ethylene, ABA, cytokinin (CK), and strigolactone (SL) having a prominent role in stress-induced leaf senescence (Abdelrahman et al., 2017). The connection between ethylene and leaf senescence is long known (Bleecker et al., 1988; Grbić and Bleecker, 1995), and numerous attempts to improve photosynthetic activity and drought performance by manipulating ethylene biosynthesis have been published in dicots (John et al., 1995) and cereal plants (Young et al., 2004). The first biotechnologically produced plant ever to reach the market with improved drought resistance due to reduced ethylene sensitivity and delayed senescence was produced by Verdeca and named HB4^®^ Drought Tolerance Soybeans (Bergau, 2019). HB4 is a modified version of the homeodomain-leucine zipper (HD-zip) transcription factor (TF) *HaHB4* from sunflower (*Helianthus annuus*). It is expressed under the control of the native soybean *HaHB4* promoter, which is stress inducible (Waltz, 2015). Although *HaHB4* does not have conserved homologs in *Arabidopsis*, upon ectopically expressing *HaHB4* in this model species, it was discovered that the TF acts at the intersection between the jasmonic acid and ethylene pathways (Dezar et al., 2005; Manavella et al., 2008). Interestingly, *HB4*-expressing soybean has increased yield in both water-limited and well-watered conditions. As shown in extensive field trials, this same gene confers similar drought tolerance properties without yield penalties when transferred to bread wheat (Gonzalez et al., 2019), with the transgenic wheat having an unaltered quality and nutritional content when compared with its parental non-transgenic variety Cadenza (Ayala et al., 2019, **Figure 2C**). As such, it is likely that the *HB4* cassette could confer drought resistance to other cereals. It is worth pointing out that the success of *HB4* is due to the exploitation of drought-responsive promoters rather than of constitutive strong promoters.

Using a rather different approach, Monsanto expressed the bacterial cold shock protein B (CSPB) under the control of the constitutive rice *ACTIN1* promoter. The expressed CSPB protein bears RNA-binding motifs named cold shock domains (CSDs) that act as RNA chaperones and regulate translational activity. In the analyzed transgenic plants, chlorophyll content and photosynthetic rates were improved (Castiglioni et al., 2008). These transgenic plants were tested in 3-year field trials in two different locations, and yields were on average 6% higher than for the control plants in water-limited conditions (**Figure 2B**). Although the molecular mechanisms are not fully understood, improved performances in water-limited conditions have been linked to a transient reduction in leaf area that leads to reduced water use and improved overall water use efficiency (WUE). This temporary dehydration avoidance does not negatively affect yield due to an improved ear partitioning, which is probably also a consequence of reduced stress exposure during vegetative growth (Nemali et al., 2015). This work led to the first biotechnologically improved crop for drought tolerance, called Genuity™ DroughtGard™ by Monsanto (**Figure 2**, event code MON-87460-4, ISAAA, 2019). Even though this result was achieved by the constitutive expression of a bacterial TF, we speculate that leaf-specific or meristem-specific genes expressed in specific developmental stages could lead to similar results.

### Stomatal-Mediated Drought Responses

Stomata, which are openings on the surface of the aerial portion of plants, are enclosed by two specialized guard cells that can open and close the pore by changing their turgor pressure. Stomata are vital for CO_2_ uptake in photosynthetic organs and are finely regulated by a molecular pathway that allows plants to acquire CO_2_ while minimizing water loss. Manipulating stomatal number, size, and regulation was one of the earliest strategies adopted by scientists in attempt to produce drought-resistant plants, and recent advances in *Arabidopsis* and crops to this effect are thoroughly reviewed in Bertolino et al., 2019 (**Figure 1C**).

The main hormone signal that triggers stomatal closure in water-limited conditions is ABA (Sussmilch and McAdam, 2017). In *Arabidopsis*, expression of the *CLAVATA3/EMBRYO-SURROUNDING REGION-RELATED 25* (*CLE25*) gene is upregulated in the root vascular tissues upon drought stress. The CLE25 peptide is translocated to the leaves where it binds to BARELY ANY MERISTEM (BAM) receptors, which, in turn, induce ABA accumulation in leaves leading to stomatal closure (Takahashi et al., 2018). The manipulation of ABA sensitivity to increase stomatal responses in response to drought could help plants to survive. However, diminished photosynthetic activity due to limited CO_2_ uptake is usually detrimental to carbon assimilation and negatively impacts crop yield. In addition, water evaporation through stomatal openings prevents plants from overheating. As drought in a natural environment is likely to be accompanied by warm temperatures, reducing stomata capacity might not be a sustainable approach to enhance drought resistance while securing yield and biomass production. For instance, a series of rice mutants of the ABA receptors *pyrabactin resistance 1-like 1* (*pyl1*), *pyl4*, and *pyl6* have improved yield but are more sensitive to drought (Miao et al., 2018), a result that resonates with the improved drought resistance but reduced yield of the transgenic plants that overexpress *PYL5* (Kim et al., 2014).

In an early attempt to produce drought-resistant plants, it was observed that the constitutive expression of *AtNF-YB1* in *Arabidopsis* improved the survival rate of the transgenic plants (Nelson et al., 2007). NUCLEAR FACTOR Y (*NF-Y*) are heterotrimeric TFs that regulate multiple developmental pathways (Zhao et al., 2017), including stomatal responses *via* modulation of the ABA signaling pathway (Bi et al., 2017), with conserved functions in *Arabidopsis* and cereals during both flowering (Siriwardana et al., 2016; Goretti et al., 2017) and DE (Hwang et al., 2019). One maize homolog of *AtNF-YB1*, *ZmNF-YB2*, was constitutively expressed under the control of the rice actin 1 promoter. Maize transgenic plants showed an improved survival rate in a greenhouse experiment, confirming the functional conservation between *Arabidopsis* and maize *NF-YBs*. In field trials, the transgenic plants were also drought resistant due to a combination of higher stomatal conductance, cooler leaf temperatures, higher chlorophyll content, and delayed onset of senescence (Nelson et al., 2007). Nevertheless, even if these transgenic lines show promising results in field trials, with the best performing line having a 50% increase in yield relative to controls under severe drought conditions, these lines were never introduced to the market, maybe because the yield in well-watered conditions was negatively affected (**Figure 2A**).

The trade-off between stomatal conductance and drought resistance could be avoided by manipulating stomatal kinetics, or more precisely, by improving the speed of stomatal responses (McAusland et al., 2016). Recently, enhanced plant stomatal kinetics was achieved by expressing a synthetic, blue light–induced K^+^ channel 1 (BLINK1) under the control of the strong guard cell–specific promoter *pMYB60* (Cominelli et al., 2011). This effectively accelerated stomatal responses, producing plants that responded faster to changing light conditions. Arabidopsis WUE (i.e., the biomass per transpired water) was improved without reducing carbon fixation rates, resulting in a 2.2-fold increase in total biomass in the transgenic plants grown in water-deficit conditions when compared with the control plants (Papanatsiou et al., 2019). Whether this approach would be efficient in crops in an open field, or whether the increased biomass would correspond to a better yield, is yet to be established. Overall, engineering the physiological behavior of stomata represents a remarkable innovation in *Arabidopsis* that has yet to be applied to crops.

### Cuticular Wax Production

Aerial plant organs have an external cuticle layer of which waxes are a major component. This hydrophobic barrier physically protects the epidermis against a plethora of external factors including UV light, cold temperatures, fungal pathogens, and insects, and also regulates permeability and water loss. However, despite the fact that a number of studies in *Arabidopsis* and crops have shown a connection between drought stress and changes in cuticular wax content, composition, and morphology, many of the key genes involved in wax metabolism, regulation, and transport still need to be characterized (Xue et al., 2017; Patwari et al., 2019). Cuticular wax composition has been studied both in *Arabidopsis* and crop species; wax composition not only varies between plant species, but also between specific tissues or organs within the same plant. In the most well-studied model, the biosynthesis of cuticular waxes occurs in epidermal cells where *de novo* synthesized C16–C18 fatty acids produced in plastids are exported by acyl–acyl carrier proteins (acyl–ACP). These proteins are subsequently hydrolyzed by the fatty acyl–ACP thioesterase B (*FATB*) and the C16–C18 fatty acids are imported into the endoplasmic reticulum following activation by long-chain acyl–coenzyme A (acyl-CoA) synthetases, which are encoded by the long-chain acyl-CoA synthetase genes *LCAS1* and *LCAS2*. The carbon chains are then elongated with C2 units from the malonyl-CoA by the fatty acids elongase complex. This complex biosynthesizes C20–C34 very-long-chain fatty acids (VLCFA) that are modified *via* two different pathways, namely the alcohol-forming pathway and the alkane-forming pathway. These pathways produce the aliphatic compounds of cuticular waxes. While the alcohol-forming pathway produces very-long-chain (VLC) primary alcohols and wax esters, the alkane-forming pathway produces VLC aldehydes, VLC alkanes, secondary alcohols, and ketones (Yeats and Rose, 2013). Besides these ubiquitous wax compounds that are common to almost all plant species, there are a plethora of specialty wax compounds that vary in carbon number, terminal carbon oxidation state, and the presence and oxidation state of secondary functional groups, with about 125 different compounds identified in over 100 plant species whose biosynthetic pathways are not yet fully described (Busta and Jetter, 2018). All wax components are synthesized in the endoplasmic reticulum and need to be exported to the plasma membrane and then secreted from the cell wall of the epidermal cells where they constitute the cuticle (Fernández et al., 2016). The secretion of wax molecules from the plasma membrane to the extracellular matrix in *Arabidopsis* is known to be mediated by the ATP binding cassette (ABC) transporters, CER5 (from *eceriferum*, waxless mutants) and WBC11 (Pighin et al., 2004; Bird et al., 2007). On the other hand, the intracellular trafficking that governs the transport of wax constituents is not fully understood, but involves more than a single mechanism. *Gnom-like 1-1* and *echidna* mutants (*gnl1-1* and *ech*), which are defective in vesicle trafficking, show a decrease in surface waxes, thereby indicating that endomembrane vesicle trafficking is required in wax transport (McFarlane et al., 2014). In addition, membrane-localized lipid transfer proteins (LTPs) may be involved in wax delivery to the cuticle through the hydrophilic cell wall. In fact, *Arabidopsis LTPG1* and *LTPG2* genes have been characterized (DeBono et al., 2009; Kim et al., 2012). Novel proteins with yet unknown molecular functions involved in extracellular wax transport are also being discovered in monocots through the characterization of mutants. One such example is maize GL6 (Li et al., 2019). A comprehensive coverage of cuticular wax biosynthesis and deposition can be found in the review articles by Bernard and Joubès (2013), and Lee and Suh (2015).

Cuticular waxes can be regulated post-translationally, post-transcriptionally, and transcriptionally. In terms of post-translational regulation, the *CER9* gene, which encodes a putative E3 ubiquitin ligase, plays a role in the homeostasis of cuticular wax biosynthetic enzymes through ubiquitination and degradation of proteins in the endoplasmic reticulum. *Arabidopsis cer9* mutants showed an increase in lipid deposition and drought tolerance, suggesting that it has a negative role with regards to the regulation of cuticular wax biosynthesis (Lü et al, 2012). *CER7*, on the other hand, which encodes an exosomal exoribonuclease that was proposed to play a role in the degradation of small RNA species that negatively regulate the CER3 transcript (an enzyme involved in wax biosynthesis), is part of the post-transcriptional mechanisms of regulation. However, transcriptional mechanisms are considered to be the main regulator of wax biosynthesis (Yeats and Rose, 2013). Accordingly, most of the biotechnological approaches that have attempted to improve drought performance by manipulating cuticular wax levels focus on TFs that control the overall process rather than on overexpressing multiple components of the biosynthetic pathways. In *Arabidopsis*, overexpression of the TF WAX INDUCER1/SHINE1 (*WIN1/SHN1*) was found to activate wax biosynthesis, increase wax deposition, and confer drought resistance in a survival rate experiment (Aharoni et al., 2004; Broun et al., 2004). In studies performed in apple and mulberry, *WIN1/SHN1* homologs have been shown to have conserved functions, and therefore might similarly increase drought tolerance (Sajeevan et al., 2017; Zhang et al., 2019). In rice, overexpression of the *WIN1/SHN1* homolog *OsWR1* improves drought tolerance at the seedling stage (Wang et al., 2012). While constitutive expression of *OsWR2* dramatically increased cuticular wax deposition (48.6% in leaves) and improved dehydration avoidance, yield was negatively affected, with a reduction of 30% in seed number per panicle (Zhou et al., 2014). On the other hand, in wheat, the overexpression of the *OsWR1* ortholog *TaSHN1—*cloned from the drought-tolerant genotype RAC875—was able to improve drought tolerance in a survival experiment without an evident loss in yield under controlled conditions. These transgenic plants have an altered wax composition and a lower stomatal density (Bi et al., 2018, **Figure 1D**). *EsWAX1*, a novel TF that was isolated from the halophyte *Eutrema salsugineum*, improves cuticular deposition and drought tolerance when ectopically expressed in *Arabidopsis*, but also leads to detrimental effects on plant growth and development. However, when expressed under the control of the stress-inducible *Arabidopsis* RD29 promoter, *EsWAX1* is able to improve the rate of drought survival without causing any major negative pleiotropic effect (Zhu et al., 2014). Even if seed number or yield was not assessed, the use of drought-responsive promoters helps overcome the undesirable effects of ectopic overexpression. Another well-known TF controlling wax biosynthesis is the *Arabidopsis* ABA-responsive R2R3-type MYB TF, *MYB96*. *MYB96* is highly expressed in stem epidermal cells, is activated by drought, and binds directly to the promoter of multiple wax biosynthetic genes to upregulate their transcripts and increase wax production (Seo et al., 2011). Overexpressing *Arabidopsis MYB96* in the close relative Brassicaceae biofuel crop *Camelina sativa* led to an increase in wax biosynthesis and deposition, and also improved drought survival of the transgenic camelina plants (Lee et al., 2014). Taken together, these results show relevant advances in the quest to obtaining drought-resistant plants by manipulating cuticular wax biosynthesis. Important differences between *Arabidopsis* and crop species in terms of wax composition, localization, and quantity need to be considered when attempting to transfer drought resistance traits. Furthermore, an excessive wax production might have negative effects on plants because of the high amount of carbon resources that need to be redirected from seeds to leaves, and because of the reduced CO_2_ permeability of the wax-covered leaves. As such, it is essential that the any biotechnologically induced increase in wax production occurs in specific cell types and in response to dehydration rather than constitutively.

### Carbon Allocation

Plants are photosynthetic organisms able to fix atmospheric carbon into macromolecules essential for growth and survival. Thus, it is evident that carbon metabolism and allocation are highly regulated and this regulation has a vital role in plant resilience to stresses and crop yield. In cereals, carbon is the main determinant of crop yield, and carbohydrates from cereals are the primary source of calories in the human diet (Lafiandra et al., 2014). One of the main pathways that regulates carbon allocation in plants is the trehalose 6-phosphate (T6P)/SNF1-related/AMPK protein kinases (*SnRK1*) pathway. T6P is a nonreducing disaccharide present in trace quantities in plants, and it acts as a signal for sucrose levels. The T6P/SnRK1 pathway has been unraveled through studies in *Arabidopsis* that led to the identification and characterization of the TREHALOSE PHOSPHATE SYNTHASE (*TPS*) and TREHALOSE PHOSPHATE PHOSPHATASE (*TPP*) genes. This pathway has also been linked to auxin and ABA signaling (Paul et al., 2018). T6P is known to act as a signaling molecule during flowering, and *Arabidopsis tps1* mutants are extremely late to flower (Wahl et al., 2013). Increasing the intracellular content of the disaccharide T6P is a well-known strategy for improving drought tolerance in plants (Romero et al., 1997). While T6P is present in trace amounts in most of temperate plants, it accumulates in resurrection plants (Wingler, 2002). However, manipulating T6P levels through the expression of T6P regulatory or biosynthetic genes under the control of strong constitutive promoters significantly alters plant growth and development, and might negatively affect crop yield (Guan and Koch, 2015). In rice, the overexpression of *Escherichia coli* T6P biosynthetic genes under the control of an artificial ABA-inducible promoter derived from the rbcS (RuBisCO) leaf-specific promoter, avoided the negative effect of ectopic T6P biosynthetic gene overexpression. In a laboratory-scale experiment, rice transgenic plants were drought tolerant, with improved photosynthetic activity and reduced photo-oxidative damage under drought conditions (Garg et al., 2002). In maize, the catabolic enzyme T6P phosphatase (*TPP*) has been specifically expressed in female floral components using the rice floral promoter gene *Mads6* (MCM1, AGAMOUS, DEFICIENS, and serum response factor). This reduced the concentration of T6P in female reproductive tissues, increased the sucrose content in the whole developing spikelet, and affected the T6P/*SnRK1* regulatory pathway. Effects on drought resistance were assessed in extensive field trials, and the yield was consistently improved in well-watered, mild, and severe drought conditions, with no obvious impact on plant or ear morphology (Nuccio et al., 2015). Crop yield and stress resilience has also been increased using chemical treatments that stimulate T6P production in *Arabidopsis* and wheat. As plants are impermeable to exogenous T6P, synthetic precursors were produced and used as treatments that triggered a light-inducible endogenous production of T6P (Griffiths et al., 2016). Trehalose was also shown to accumulate in the roots of plants with augmented brassinosteroid (BR) signaling, together with other osmoprotective sugars like sucrose and raffinose. Furthermore, T6P-related gene expression was specifically upregulated in the root phloem cells (Fàbregas et al., 2018, **Figure 1E**). In turn, by mediating BR signaling, sugars act as signaling molecules in *Arabidopsis* to control different aspects of root system architecture, such as primary root elongation, lateral root development, and root directional response (Gupta et al., 2015; Zhang and He, 2015). Notably, manipulation of BR signaling results in an increase in osmoprotectant metabolites including proline, an amino acid long known for conferring drought and salinity tolerance (Kishor et al., 1995). Thus, accumulation of sugars and proline might also be a valid strategy to achieve dehydration tolerance in cereals, as shown by transgenic maize plants that constitutively express the vacuolar H^+^-pyrophosphatase (V-H^+^-PPase) gene (*TsVP*) from the halophyte *Thellungiella halophila*. In a small-scale field experiment, these transgenic plants showed a higher yield under drought conditions than the control plants (Li et al., 2008, **Figure 2E**).

Altering sugar distribution *via* the T6P pathway is a promising biotechnological approach for producing drought-tolerant plants, with the best results being obtained when manipulation is directed to specific tissues like developing reproductive structures (Nuccio et al., 2015; Oszvald et al., 2018) and seeds (Kretzschmar et al., 2015; Griffiths et al., 2016). Notably, seed-specific manipulation of T6P might increase drought tolerance as well as resistance to flooding (Kretzschmar et al., 2015). As most of the plant sugar trafficking happens through the phloem, shoot and root vascular tissues are also candidate targets for T6P manipulation (Griffiths et al., 2016; Fàbregas et al., 2018).

### Root Traits

Roots are the main plant organ dedicated to the uptake of water, and are the first place where a lack of water is perceived. As such, an abundance of studies have examined root responses to dehydration. The most relevant root traits capable of improving drought tolerance and their biotechnological applications have recently been reviewed by Koevoets et al. (2016) and by Rogers and Benfey (2015), respectively. Here, we will focus on the solutions offered by manipulation of the BR pathway, and will provide a brief overview on the most promising biotechnological strategies aimed at improving drought resistance through manipulating root-related traits. BRs are a class of plant hormones that are widely involved in plant growth and development, as well as in stress responses. Along with other plant hormones, BRs play a key role in root growth. As BR levels are finely regulated to permit proper root development, BR metabolism and signaling are clear targets for the manipulation of root responses (Singh and Savaldi-Goldstein, 2015; Planas-Riverola et al., 2019). Indeed, exogenous application of BRs has been extensively tested on a variety of crops with variable outcomes (Khripach et al., 2000). However, from a genetic perspective, the only BR-related mutant widely used in agriculture is the barley *uzu* mutant, which carries a single amino acid substitution in the BR receptor *HvBRI1*, homolog of the Arabidopsis BR receptor Brassinosteroid insensitive-1 (BRI1) and displays a semi-dwarf phenotype (Chono et al., 2003). Recently, the triple mutant of *wrky46*, *wrky54*, and *wrky70*—positive regulators of BR signaling *Arabidopsis* group III WRKY TFs—was shown to be drought resistant. Due to a significant upregulation and downregulation of dehydration-induced and dehydration-repressed genes, respectively, these TFs operate as negative regulators of drought tolerance (Chen et al., 2017). BR biosynthetic dwarf and semi-dwarf mutants were also shown to be drought tolerant (Beste et al., 2011). Somehow, contrasting with these results, it has recently been demonstrated that the overexpression of vascular-specific BR receptor BRI1-LIKE 3 (*BRL3*) increases the survival rate of *Arabidopsis* plants exposed to severe drought stress. Interestingly, these transgenic plants do not show reduced growth, which is typically associated with drought-resistant BR mutants, and retain the same RWC as wild-type plants. As previously mentioned, these transgenic plants displayed an osmoprotectant signature (proline, trehalose, sucrose, and raffinose) in response to drought, with the corresponding biosynthetic and metabolic genes upregulated in the root phloem. This might suggest that BRs are involved in dehydration tolerance as well as in dehydration avoidance (Fàbregas et al., 2018, **Figure 1F**). BRs are also involved in hydrotropism, with the receptors BRI1-LIKE 1 (BRL1) and BRL3 having a prominent role that is independent of the pathway. Interestingly, BRL3 is structurally and functionally very similar to BRI1, but its expression is confined to the root stem cell niche while that of BRI1 is ubiquitously found in the root. This suggest that the BR-related drought responses in roots could be led by BR receptors in specific cells, such as the root meristematic region and vascular tissues (Fàbregas et al., 2018). In crops, increments in the BR biosynthetic pathway were shown to improve both stress tolerance—including dehydration and heat stress—and seed yield in the oil crop *Brassica napus* (Sahni et al., 2016). Furthermore, in wheat, the overexpression of the BES/BZR family TF gene *TaBZR2*, a positive regulator of BR signaling, enhanced the expression of wheat glutathione S-transferase 1, *TaGST1*. These transgenic plants showed an increase in ROS scavenging and a drought-resistant phenotype without being dwarf (Cui et al., 2019). The seemingly opposed behavior of BR-engineered plants could be partly explained by the drought stress experimental setup. Most BR biosynthetic and signaling mutants exhibit an evident dwarf phenotype. However, in drought survival experiments, dwarf plants often show a passive drought resistance phenotype, and it is challenging to dissect whether a phenotype is due to a direct genetic effect on drought-related gene expression, or if it is a dehydration avoidance mechanism due to limited water consumption. Still, the manipulation of BR pathways retains its full potential with respect to the development of stress-tolerant varieties, particularly if directed to specific cell types to avoid unnecessary ectopic expression, and because of the involvement of these pathways in many agriculturally relevant traits such as grain shape and size, cell elongation and plant height, leaf angle, and root development (Espinosa-Ruiz et al., 2017; Martins et al., 2017; Tong and Chu, 2018). Unfortunately, translating root responses from *Arabidopsis* to cereals is particularly challenging as the root system and architecture differ greatly among plant species. Nevertheless, interesting results were obtained in cereals by engineering root responses. In rice, expressing the TF *OsNAC5* under the control of the root-specific promoter *RCc3* (Xu et al., 1995) improved drought resistance by increasing root diameter. Specifically, enlarged metaxylematic vascular tissues permitted the transgenic plants to have a better water flux. The use of a tissue-specific promoter was paramount for the success of this experimental approach. Indeed, when expressed under a constitutively strong promoter, the same *OsNAC5* was not able to increase yield under drought because of a reduction in the grain filling rate (Jeong et al., 2013, **Figure 2F**). Another example is the rice NAC family, which is well known for its effect on root architecture and stress responses. Several genes of this family have been overexpressed (*OsNAC9*, Redillas et al., 2012) or expressed under the control of the root-specific promoter *RCc3* (*OsNAC10*, Jeong et al., 2010), with similar effects on drought and stress tolerance. Another superfamily of stress-related TFs, the APETALA2/ethylene responsive element binding factors (AP2/ERF), has been extensively studied in attempt to enhance root traits and achieve improved drought tolerance. AP2/ERF TFs participate in drought and cold stress responses (Shinozaki et al., 2003) and the overexpression of AP2/ERF genes increases stress tolerance in *Arabidopsis* (Haake et al., 2002). The *Arabidopsis* HARDY (HRD) gene, a AP2/ERF TF, was identified as a dominant mutant with increased root density, and its ectopic expression was able to improve the survival rate of both *Arabidopsis* and rice (Karaba et al., 2007). Ectopic HRD expression also alters leaf morphology, with thicker deep-green leaves in *Arabidopsis* and increased shoot biomass in rice contributing to an improved WUE of the transgenic plants. However, the increased WUE was measured as an increase in biomass and no data regarding seed production and yield were reported. Similar promising results were obtained in the fodder dicot *Trifolium alexandrinum* by constitutively expressing *Arabidopsis* HRD. This transgenic plant had a larger biomass in drought and salt stress conditions as tested in a controlled environment and in field trials (Abogadallah et al., 2011). In bread wheat, the expression of the *Arabidopsis* AP2/ERF TF DREB1A gene under the control of the stress-inducible RD29A promoter delayed leaf wilting in a water withholding experiment in a controlled environment (Pellegrineschi et al., 2004). In rice, a root-specific drought-responsive AP2/ERF TF OsERF71 was cloned and expressed either in the whole plant using the rice GOS2 promoter (de Pater et al., 1992), or specifically in the roots using the *RCc3* promoter. Both transgenic lines proved to be drought resistant. In addition, the root-specific expression was able to improve grain yield in drought conditions. OsERF71 can bind to the promoter of the key lignin biosynthesis gene *OsCINNAMOYL-COENZYME A REDUCTASE1*, and it was proposed that changes in cell wall and root structure were the basis of the drought-resistant phenotype (Lee et al., 2016). However, OsERF71 overexpression has a much wider impact on plant transcriptional regulation; it induces the oxidative response and DNA replication, and reduces photosynthesis, thereby diverting more resources toward survival-related mechanisms (Ahn et al., 2017). Native OsERF71 expression is induced by ABA, and in turn regulates the expression of ABA-related and proline biosynthesis genes in drought stress conditions (Li et al, 2018b). Another set of studies aimed at improving abiotic stress tolerance in rice found that the expression of barley late embryogenesis abundant (LEA) protein HVA1 under the control of a synthetic ABA-inducible promoter increased the root system expansion. LEA proteins are encoded by stress-responsive genes, and barley HVA1 and its rice homolog LEA3 are well known for being regulated in roots in response to ABA, salt, and abiotic stresses. LEA proteins could work as osmoprotectants by maintaining cell functionality and conferring dehydration tolerance. In this rice study, the synthetic promoter *3xABRC321*, which carries a series of ABA-responsive elements, drove the expression of HVA1 in response to abiotic stress specifically in the root apical meristem and lateral root primordia. In turn, both primary and secondary root growth was significantly promoted through an auxin-dependent process. These transgenic rice plants showed a better WUE and abiotic stress tolerance in a small-scale field trial (Chen et al., 2015). In rice, QTL *DEEPER ROOTING 1* (*DRO1*), which controls root angle, was studied using shallow- and deep-rooting cultivars, and was identified by developing a near-isogenic line homozygous for the allele conferring the deep-root trait. DRO1 is expressed in the root meristematic region, is controlled by auxins, and regulates the gravitropic response. The DRO1 deep-root allele from the cultivar Kinandang Patong (*DRO1-kp*) contains a 1-bp deletion that results in a premature stop codon, shortening the C-terminal domain of the protein that it encodes. *DRO-kp* lines have an enhanced gravitropic response that leads to deeper roots and drought avoidance, and ultimately improves rice yield under drought conditions (Uga et al., 2013). Furthermore, as shown in paddy field trials, the yield of *DRO-kp* lines is also improved in normal growth conditions (Arai-Sanoh et al., 2014). Although DRO1 does not have a clear homolog in *Arabidopsis*, it has homologs in other monocots like maize. The C-terminal position of the stop codon in the *DRO1-kp* alleles makes DRO1 an ideal target for CRISPR-based gene targeting.

## Challenges and Future Perspectives

### Genome Editing for Drought-Resistant Crops

During the past 10 years, genome editing technologies like zinc fingers nucleases (ZFNs), transcription activator–like effectors nucleases (TALENs), and homing meganucleases (also known as meganucleases) have enabled scientists to produce targeted genetic modifications in organisms of choice (Arnould et al., 2011; Bogdanove and Voytas, 2011; Carroll, 2011). Innovative cloning approaches like the Golden Gate system made the assembly of these tools more straightforward (Cermak et al., 2011); however, genome editing protocols were still relatively time consuming and labor intensive. With the advent of the engineered clustered regularly interspaced short palindromic repeats/CRISPR-associated protein 9 (CRISPR/Cas9) system to perform targeted mutagenesis, genome editing became accessible to most research laboratories (Gasiunas et al., 2012; Jinek et al., 2012). In CRISPR-based genome editing, specificity to the target sequence is conferred by a programmable short fragment of RNA called guide RNA (gRNA), and the Cas9 protein itself does not require any structural modification to change target recognition. This is similar to what happens in the case of ZFNs and TALENs (Cong et al., 2013). CRISPR/Cas9 is derived from the bacterial immune system against viral infections. It was first observed by sequencing the DNA of *E. coli*, where a short series of DNA repeats are separated by spacer sequences (Ishino et al., 1987). The spacer sequences are DNA from viral invaders that the bacteria store as a sort of immune memory (Mojica et al., 2000; Mojica et al., 2005). These CRISPR sequences are transcribed and processed into short CRISPR RNAs (crRNAs), which are composed of a variable spacer portion and a conserved protospacer repeat, and subsequently associated with a trans-activating CRISPR RNA (tracrRNA). The ribonucleoprotein complex composed by Cas9, crRNA, and tracrRNA are finally directed toward the invading DNA complementary to the spacer (Jansen et al., 2002; Bolotin et al., 2005; Pourcel et al., 2005). This system was engineered in such a way that the crRNA and tracrRNA were fused together in a unique fragment, the gRNA. Simply modifying the 20 nucleotides corresponding to the spacer is sufficient to target the Cas9 to a sequence of choice (Cong et al., 2013).

The implementation of CRISPR-based genome editing technologies in plant science opened up a wealth of opportunities to plant scientists and plant breeders alike (Li et al., 2013; Shan et al., 2013). The most straightforward application of CRISPR/Cas9 is the production of out-of-frame *loss-of-function* mutants. Interestingly, *loss-of-function* mutations are the most frequent kind of genomic modification that happened during the domestication of crops. In fact, from a genetic perspective, crop domestication was achieved by stacking *loss-of-function* mutants in key genes controlling traits like seed shattering, flowering time, seed color, or size (Meyer and Purugganan, 2013). By targeting these genes, scientists have been able to swiftly retrace thousands of years of crop improvement in a process known as *de novo* domestication (Zsögön et al., 2017; Li et al., 2018c). Ideally, this approach could assist with the rapid improvement of highly resilient, locally adapted species to obtain new commercially relevant crops that still retain the unaltered stress resistance characteristics of their wild-type relatives. For some crops, *de novo* domestication could be more efficient than breeding into modern commercial varieties since stress resistance traits, often controlled by multiple, sometimes unknown, genes, have been lost during crop domestication.

**Figure 3:**
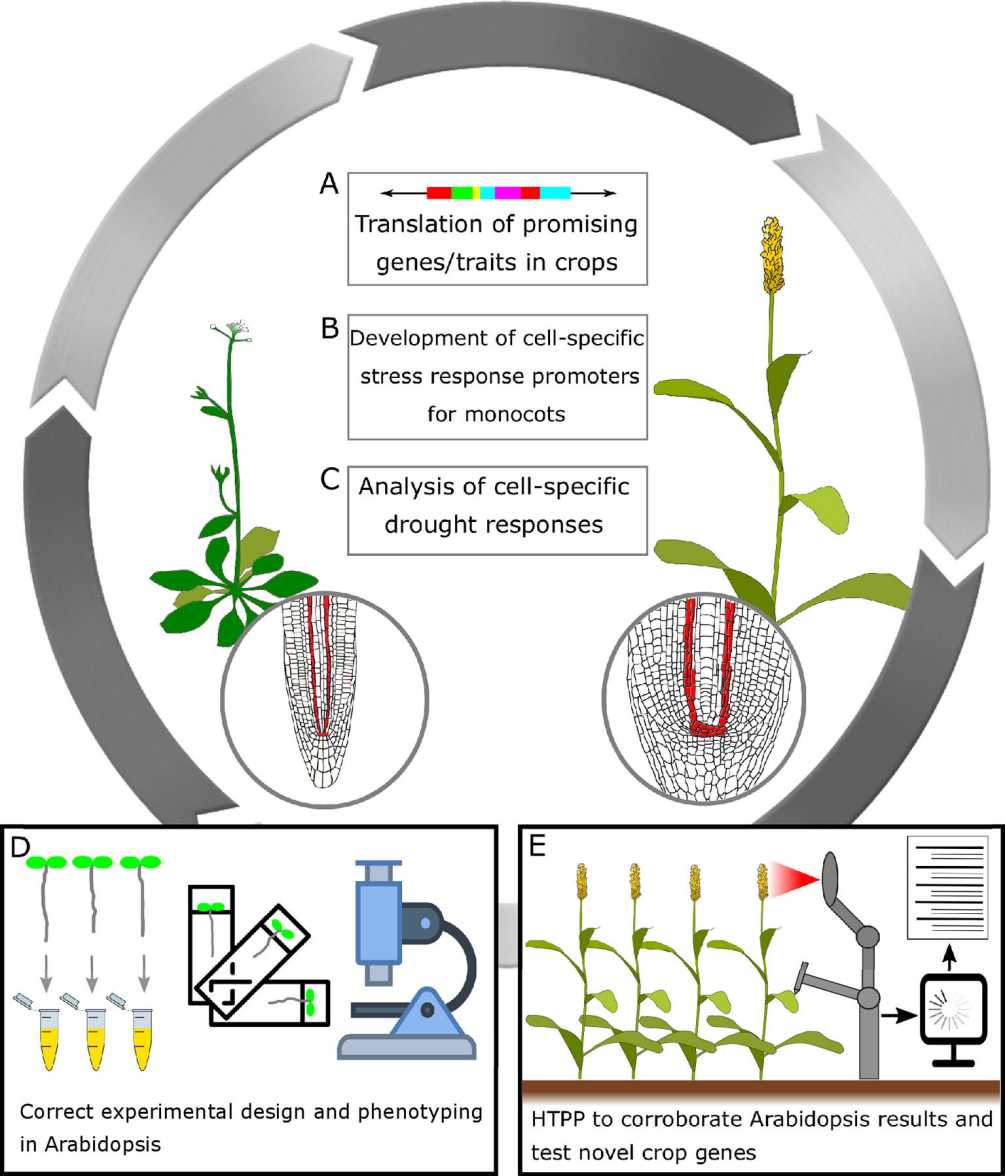
A general frame for translating research in *Arabidopsis thaliana* to crops to improve drought performance in cereals. **(A)** Translation of promising genes/traits in crops: In recent years, the improvement of genome editing technologies has enabled targeted genetic modifications of organisms of choice, and has opened up a wealth of opportunities to plant scientists and plant breeders alike. Genome editing technologies might also help drive the development of more efficient crop transformation methods. **(B)** Development of cell-specific stress response promoters for monocots: It has been shown that the use of moderate constitutive, tissue-specific, and drought-responsive promoters could limit unintended pleiotropic effects in terms of growth or yield penalty while maintaining the improved trait (Waltz, 2015; Fàbregas et al., 2018; Papanatsiou et al., 2019). **(C)** Analysis of cell-specific drought responses: As of today, cell-specific promoters available for cereals are limited. By performing transcriptomic studies coupled to FACS (fluorescence-activated cell sorting) in the crops of interest, it could be possible to identify novel cell-specific promoters. Subsequently, these promoters could be exploited and introduced into crops. Drought stress research will greatly benefit from tissue-specific -omics. **(D)** Correct experimental design and phenotyping in *Arabidopsis*: Many *Arabidopsis* drought-stress experiments are performed without recording traits such as yield, plant biomass, relative water content (RWC), etc. Moreover, near-lethal conditions do not reflect in crop performances in open fields. Future studies should consider all these aspects. Recording more detailed data, plus validating these promising results in crops, should be a priority for any research group working in *Arabidopsis*. **(E)** High-throughput plant phenotyping (HTPP) to corroborate *Arabidopsis* results and test novel crop genes: HTPP for drought has been implemented in *Arabidopsis*, and it is in development for many crops. HTTP will help increase and improve the reproducibility and quality of data from drought adaptation studies. The widespread adoption of HTTP platforms could represent a valid intermediate step between laboratory conditions and open, large-scale field trials.

Genome editing technologies might also help speed up molecular breeding and crop improvement for so-called orphan crops, plants that are critical to local food security but are less relevant on a global scale (e.g., sweet potato, chickpea, or sorghum) (Lemmon et al., 2018; Li et al., 2018a). The rationale behind these approaches is similar to that of the *de novo* domestication strategy: improving a resilient, locally adapted, and highly specialized crop might provide better results than attempting to restore stress tolerance in currently used elite varieties in which complex multigenic traits were lost over the domestication process (Khan et al., 2019). One of the main limits to genome editing in crops is plant transformation efficiency, which hampers the delivery of genome editing material into the target cells. For most wild or orphan species, genetic transformation has never been attempted, and in the remaining cases, current protocols are only efficient for a small subset of the lab amenable varieties. Nonetheless, the high potential of genome editing in plant sciences is driving the development of more efficient crop transformation methods (**Figure 3A**).

DuPont Pioneer scientists have successfully used CRISPR/Cas9 to engineer drought tolerance by swapping the native promoter of the ARGOS8 gene for the promoter of maize *GOS2*. The maize GOS2 promoter was identified from the rice homolog GOS2 (de Pater et al., 1992), and in this case conferred a moderate ubiquitous expression to ARGOS8. In field trials, these *cis-*genic lines showed increased yield under drought conditions (Shi et al., 2017). ARGOS were previously studied as negative regulators of ethylene signaling in both *Arabidopsis* and maize. The constitutive overexpression of *ZmARGOS1*, *ZmARGOS8*, and Arabidopsis *ARGOS* were shown to decrease ethylene sensitivity in transgenic *Arabidopsis* plants (Shi et al., 2015). *ARGOS* genes and their molecular mechanisms are conserved in both model and crop species. However, constitutively high expression had a negative effect in cool and high humidity conditions (Guo et al., 2014). The use of the maize GOS2 promoter enabled a moderate constitutive expression that delivered drought resistance without affecting yield in normal or humid conditions (Shi et al., 2017). This work shows that combining genome editing with promoters of tailored activity levels can provide the basis for successfully producing drought-resistant crops (**Figure 2D**).

### Tissue-Specific Promoters to Drive Drought Tolerance

Basic plant science research, as well as most traditional breeding and biotechnological approaches, are based on *loss-of-function* or *gain-of-function* mutants, or on the constitutive expression of a gene conferring a certain trait. As an example, mutations in the MILDEW RESISTANCE LOCUS O (*Mlo*) genes confer broad-spectrum resistance against fungal pathogens to a large number of plant species including major cereals like wheat and barley (Kusch and Panstruga, 2017). Similarly, *Bt* crops constitutively expressing bacterial toxins from *Bacillus thuringiensis* are used worldwide to protect crops from pathogens (ISAAA Brief, 2017). However, improving resistance to abiotic stresses does not seems to follow the same pattern (Todaka et al., 2015). Dehydration avoidance based on a reduction in size or density of stomata comes with a growth or yield penalty (Bertolino et al., 2019). Manipulating hormone signaling pathways by means of ectopic overexpression of its components, or alternatively, by knocking them out, often has an undesired pleiotropic negative effect on overall plant growth and development. In contrast, it has already been shown in maize that the use of moderate constitutive promoters instead of strong promoters could limit this undesired effect while maintaining the improved trait (Shi et al., 2017) (**Figure 2B**).

The use of tissue-specific promoters to drive gene expression in particular cells upon drought stress stands as a promising solution to break the deadlock between drought resistance and yield penalties. Several emerging studies show that when a tissue-specific promoter is used, it is possible to reap the benefits of the expressed genes while avoiding any major alteration to overall plant phenotype. This is the case for the guard cell–specific promoter *pMYB60*, which was used to express the synthetic protein BLINK1 in stomata (Papanatsiou et al., 2019), and the *rbcS* leaf-specific promoter that was used to express T6P biosynthetic genes in leaves (Garg et al., 2002). Similarly, the use of stress-inducible promoters has proven to be an effective strategy to improve drought performances without penalizing yield in soybean and wheat (Waltz, 2015; Gonzalez et al., 2019). Importantly, these transgenic plants were tested extensively in field trials, and the transgenic HB4 soybean is one of the very few biotechnologically improved drought-resistant plants ever to be introduced on the market (ISAAA, 2019). Our recent findings revealed that the overexpression of the vascular *AtBRL3* receptor confers drought tolerance without any evidence of growth penalty (Fàbregas et al., 2018), and thereby opens up new and exciting possibilities to address the societal demand for producing “more crop per drop” and to ensure global food security goals in the upcoming years. It would be interesting to assess what the effect would be of expressing the drought resistance genes that have been isolated over the years under the control of a promoter that is both stress-inducible and cell-specific, similarly to what was demonstrated in rice by expressing the barley LEA protein HVA1 under the control of a synthetic promoter (Chen et al., 2015).

The main drawback of this approach is the limited availability of crop promoters that allow such specific gene expression. This hurdle could be overcome by performing transcriptomics in crops under normal and stress conditions that accurately differentiate between tissues. For example, in a study performed in rice, metabolomic and transcriptomic profiling was performed using samples representing developed leaves and the SAM region exposed to progressively harsher drought conditions. Different responses from the plant were recorded. Mild stress induced stomatal responses, decreased auxin and CK levels, and thus plant growth, while more severe stress resulted in the production of ABA and the remobilization of sugars (Todaka et al., 2017). Differentially regulated genes identified by this and similar studies will hopefully lead to the isolation of tissue-specific, drought-responsive promoters. Species-specific responses were also observed in this study; in contrast to what previously reported in *Arabidopsis*, moderate drought stress did not activate ethylene-responsive genes in rice (Skirycz et al., 2011). Thus, as drought response pathways might differ significantly from those of *Arabidopsis*, it will be important to perform transcriptomic studies directly on the crops of interest. This might also help identify novel, species-specific components of the drought response.

Once a sufficient number of promoters are identified and tested in crops, a virtuous circle might be triggered in which transgenic cereals expressing tissue-specific markers would enable tissue-specific transcriptomics. This in turn could lead to the discovery of novel, cell type– and response-specific promoters that might provide innovative solutions to plant biotechnologists. Using fluorescence-activated cell sorting (FACS), a large number of plant seedlings expressing cell type–specific fluorescent markers could be grown in the desired experimental conditions, and then protoplasts prepared and sorted by flow cytometry to collect cells for -omics studies (Birnbaum et al., 2005). In *Arabidopsis*, methods to perform RNAseq transcriptomics with as few as 40 cells isolated using FACS have been developed (Clark et al., 2018). In fact, efficient protoplast preparation protocols that enable quick preparation and sorting of protoplasts—avoiding major transcriptional changes—are already available for rice (Zhang et al., 2011), and FACS has already been used to study stress responses in this crop (Evrard et al., 2012). Similarly, a protocol to prepare protoplasts for FACS has already been developed for maize (Ortiz-Ramirez et al., 2018). An alternative approach for single-cell isolation is INTACT (isolation of nuclei tagged in specific cell types), where cell type–specific nuclei are isolated by affinity purifying a transgenic label targeted to the cell nucleus (Deal and Henikoff, 2011). INTACT does not require specialized instruments for cell sorting and might be preferred for chromatin studies (Deal and Henikoff, 2010). However, so far it has been tested exclusively on the model plant *Arabidopsis*. Both methods need transgenic plants expressing a fluorescent marker or a nuclear protein label. In cereals, depending on the species, plant transformation might still be challenging and a protoplast preparation protocol might be tedious, time consuming, and may significantly alter the transcriptional responses. In fact, so far most of the tissue-specific studies performed in monocots have used laser capture microdissection to isolate tissues. However, it is expected that cereal-adapted protocols will soon be developed to enable advanced transcriptomics. Regardless of the method of choice, drought stress research in cereals will greatly benefit from tissue-specific -omics, especially considering how just a few relevant cell types appear to control major responses (Efroni and Birnbaum, 2016) (**Figure 3C**). In *Arabidopsis*, this field of research is quickly developing and has led to even more exciting approaches that allow high-throughput single-cell RNA sequencing (scRNA-seq). In scRNA-seq, protoplasted cells are encapsulated in individual droplets and each cell transcriptome is individually analyzed. Cells are then bioinformatically organized into tissues and cell types based on the presence of marker genes, which in the case of *Arabidopsis*, are well established for each cell type. In turn, novel, highly specific marker genes can be identified. This approach is called Drop-seq, and while it was initially developed for animal cell studies (Macosko et al., 2015), it has been adapted for *Arabidopsis* root cells to study development (Denyer et al., 2019; Ryu et al., 2019) and responses to treatments (Shulse et al., 2019).

### Cereal Transformation

With the notable exceptions of rice and maize, for which transformation efficiencies can reach up to 100% and 70%, respectively, plant transformation is notoriously challenging in cereal crops and involves time-consuming protocols that often need to be performed by highly skilled technicians (Singh and Prasad, 2016). Even when efficient protocols have been developed for the species of interest, high transformation efficiency is usually confined to just a few laboratory varieties (Harwood, 2012). In addition, as these varieties are often obsolete, an introgression program into current elite varieties must follow, thereby further hampering applicability of plant research in plant breeding.

The advent of genome editing is rapidly altering this scenario. The wealth of opportunities that are opening up as a result of the rapidly advancing CRISPR-based technologies are driving a new wave of technological development in plant transformation (Kausch et al., 2019). A recent breakthrough by DuPont Pioneer (now Corteva) scientists was achieved by applying morphogenic regulator genes like BABYBOOM (*BBM*) and WUSCHEL (*WUS*) as transformation adjuvants (Lowe et al., 2016). This ultimately optimized the idea of using growth-stimulating genes in plant transformation (Ebinuma et al., 1997). Co-delivery of *BBM* and *WUS*, either as proteins or coding sequences, together with the target sequences seems to considerably improve transformation efficiency in a number of notoriously recalcitrant species like sorghum and sugarcane, as well as in elite varieties of maize (Lowe et al., 2016; Mookkan et al., 2017).

Scientists at the University of California, Berkeley (USA), developed an interesting approach to plant transformation, which is distinct from both *Agrobacterium*- and biolistic-based systems. In this novel approach, a DNA delivery system makes use of carbon nanotubes (Demirer et al., 2019). While transformation was achieved only transiently in leaves or protoplasts of the target plants, this method has the notable advantage of being genotype independent. The lack of transgene integration could actually represent a critical advantage when CRISPR/Cas9 genome editing is involved. Indeed, it has been proven that transient expression of Cas9 and gRNAs in the target cells is sufficient to produce stable and heritable edits (Zhang et al., 2016). The advantage of this approach is that the first generation of mutants after transformation can carry the desired modifications, with no need to segregate the CRISPR material. This makes it attractive, especially for use in crops that are difficult or impossible to cross. Even though this approach is still in its proof-of-concept stage, it represents an innovative and potentially groundbreaking technology.

### High-Throughput Plant Phenotyping for Drought Traits

Despite the vast amount of information that has been reported to date regarding drought in Arabidopsis, Bayer’s (then Monsanto) DroughtGard^®^ maize, Verdeca’s HB4 soybean and wheat, and Indonesian Perkebunan Nusantara’s NXI-4T sugarcane are the only biotechnologically improved drought-resistant crops ever introduced onto the market (Nuccio et al., 2018). This gap can only partially be explained by societal and market opposition to genetically modified (GM) crops. The main hurdle in translating *Arabidopsis*-developed drought-resistant traits into crops is the fact that most of the laboratory-scale, *Arabidopsis*-based drought studies have limited data collection and phenotyping (Blum, 2014). As a general example, most of the *Arabidopsis* drought-stress experiments are performed by suspending irrigation for an extended time (12–21 days) followed by re-watering. The survival rate (live/dead plants) is then measured a few days (2–7) after. In this set of experiments, data regarding soil moisture, plant biomass, RWC, and seed yield are often not recorded. These dehydration survival experiments in near lethal conditions do not often reflect in crop performances in open fields, and do not relate to improved yield under drought or normal conditions (**Figure 3D**). Furthermore, plants have evolved survival traits to maximize fitness when growth conditions are not ideal, often by decreasing total seed number to ensure the full viability of a limited number of seeds. However, the ultimate goal of plant breeding is to increase or secure plant yield and production, not plant survival (Skirycz et al., 2011; Blum and Tuberosa, 2018). Future experimental planning with more thorough data collection beyond mere survival rate and that includes yield evaluations might overcome this limitation (Zhou et al., 2016). Validating the most promising results in a crop model should become the priority for any research group that works in *Arabidopsis*. Alternatively, collaborations between *Arabidopsis* and crop scientists and/or plant breeders should be established to streamline the translation of innovative biotechnological approaches for use in agricultural science. Furthermore, industrial partnerships with plant breeding companies or seed companies could provide both the means and the expertise to test engineered plants in extensive field trials, tests which would otherwise prove unpractical or financially unsustainable in most research laboratories.

In parallel, the advent of high-throughput plant phenotyping (HTPP) platforms and the establishment of research infrastructure networks like the EPPN2020 (https://eppn2020.plant-phenotyping.eu/) will definitively help to increase and improve the reproducibility and quality and quantity of data from drought adaptation studies. HTPP for drought responses has been implemented for *Arabidopsis* (Granier et al., 2006; Jansen et al., 2009; Skirycz et al., 2011; Fujita et al., 2018) and applied to drought research (Rosa et al., 2019). Outdoor or greenhouse HTPP facilities to study drought performances are being developed for crops (Fahlgren et al., 2015). These facilities are usually capable of capturing multispectral images of the plants, weighing the pots as a gravimetric, indirect measure of soil moisture, and differentially irrigating the plants to allow drought-exposed and control plants to be placed in the same environment. Generally, these systems are based either on a robotized apparatus that moves around the plants performing measurements and irrigation (Gosseau et al., 2019), or exploits a mobile device that scans plants for images while the pots stand on scales (Vadez et al., 2015). Alternatively, the pots are arranged on a conveyor belt system that transports the plants to watering or imaging stations, like in the APPP systems at IPK Gatersleben, Germany (Junker et al., 2014). As reproducibility of results (Editorial, 2016) might deter plant breeders and investors, a widespread adoption of HTPP could not only support the validity of experimental results, but also represent a valid intermediate step before large-scale field trials, especially when a series of different genotypes with comparable drought performances are involved (**Figure 3E**).

## Summary

In this review, we highlight that many physiological mechanisms underlying drought-resistance traits are conserved between *Arabidopsis* and crops. DE, control of flowering time, stomatal responses, T6P pathways, and some root traits are highly conserved among plants. Therefore, *Arabidopsis* is an excellent model to test drought responsive strategies. Still, when studies performed in *Arabidopsis* reveal interesting agronomic potential, these results should promptly be translated into laboratory-amenable cereal crops like rice.

On the other hand, traits like cuticular waxes, senescence, and stay-green might have significant differences that would need to be carefully assessed using a species-by-species approach. Nonetheless, *Arabidopsis* could still provide a useful heterologous system to test novel genes discovered in cereal species and their relative molecular responses.

As a general frame to help translate research in *Arabidopsis* into crops, and with the ultimate goal of improving drought performance in cereals, we suggest the following measures to be adopted: a) use an accurate experimental design in *Arabidopsis*; b) timely translate promising genes/traits in model crops (i.e., rice); c) include HTPP to corroborate *Arabidopsis* results and to test novel crop genotypes; d) investigate tissue- and cell type–specific drought responses; and e) clone tissue- and cell type–specific, stress-responsive promoters for monocots and make available them to the entire scientific community.

It is crucial to strengthen the bridges between *Arabidopsis* and crop scientists. Moreover, the coordination of research groups and institutes working with *Arabidopsis* and crop species at the same time will be important in facilitating this process. In addition, academia–industry partnerships could prove instrumental not only for rapidly scaling up promising results, but also for designing potential drought-resistant strategies that might have a high impact on global agriculture.

## Author Contributions

DM and AC-D outlined and wrote the manuscript together with AR-M (designed the figures), JF-M, and DB-E. All authors reviewed and edited the manuscript.

## Funding

AIC-D is a recipient of a BIO2016-78150-P grant funded by the Spanish Ministry of Economy and Competitiveness and Agencia Estatal de Investigación (MINECO/AEI) and Fondo Europeo de Desarrollo Regional (FEDER), and a European Research Council, ERC Consolidator Grant (ERC-2015-CoG – 683163). JBF-M is supported by the grant 2017SGR718 from Secretaria d’Universitats i Recerca del Departament d’Empresa i Coneixement de la Generalitat de Catalunya and by the ERC- 2015-CoG – 683163 granted to the AIC-D laboratory. AR-M is a predoctoral fellow from Fundación Tatiana Pérez de Guzmán el Bueno. DB-E and DM are funded by the ERC-2015-CoG – 683163 granted to the AIC-D laboratory. This project has received funding from the European Research Council (ERC) under the European Union’s Horizon 2020 research and innovation programme (Grant Agreement No 683163). This work was supported by the CERCA Programme from the Generalitat de Catalunya. We acknowledge financial support from the Spanish Ministry of Economy and Competitiveness (MINECO), through the “Severo Ochoa Programme for Centres of Excellence in R&D” 2016-2019 (SEV-2015-0533).

## Conflict of Interest

The authors declare that the research was conducted in the absence of any commercial or financial relationships that could be construed as a potential conflict of interest.
